# Cancer Risk and *GSTM1* and *GSTT1* Polymorphisms

**DOI:** 10.1289/ehp.0900829

**Published:** 2009-07

**Authors:** Francesco Cetta, Armand Dhamo, Laura Moltoni, Rosalia Zangari

**Affiliations:** PAT Geriatric Institute, Milan, Italy, E-mail: cetta@unisi.it; Department of Surgery, Research Doctorate in Oncology and Genetics, University of Siena, Siena, Italy

Rossi et al. (2009) stated in their conclusion that *“GSTM1* [glutathione *S*-transferase M1] and *GSTT1* [glutathione *S*-transferase theta 1]polymorphisms [as all individual polymorphisms] … are not expected to have a dramatic influence on baseline CA [chromosomal aberration] or overall cancer risk.”

We agree with these statements from a general point of view. However, it is one thing to suggest that an evident pathologic marker, such as CA frequency in peripheral lymphocytes, could be an expression of cancer (like elevated carcinoembryonic antigen or other biomarkers) and another to exclude any influence of a genetic polymorphism on the occurrence of a specific type of cancer on the basis of a study that is basically not suitable to answer the question.

We will not address the advantages of the Bayesian approach versus the classic frequentist model. However, as clinicians, we would like to comment on epidemiologic studies on cancer, in particular those concerning the possible effects of complex causative factors such as environmental pollution. We also will discuss issues concerning patients and outcomes of the article by Rossi et al. (2009). In particular, we will focus on issues that are often considered by epidemiologists and those interested in statistical analysis to be pathophysiologic or pathogenetic details, but are, on the contrary, basic issues for those examining clinical and pathological findings.

Although the combination of bone and skin cancers (cancers that originate from different tissues and are related to completely different pathogenetic agents and pathophysiologic mechanisms) could be acceptable from a statistical point of view, this practice creates a methodologic bias from pathophysiologic and pathogenetic points of view. Because Rossi et al. (2009) included a large number of bone and skin cancer cases in their study (*n* = 20 in their Table 2), it is of paramount importance to state whether cytochrome P451 A1 (CYP1A1) is a basic factor in the occurrence of these cancers. For lung and respiratory tract cancer, the role of CYP1A1 has been tested; however, it is not appropriate to use these polymorphisms, which are specific for the metabolism of some xenobiotics, as a marker of all cancers.

Our team has long been involved in the detection of cause and effect relationships between presumed causative factors and cancer, in particular, concerning the relative role of inherited predisposition and environmental factors, the relative impact of intrinsic toxicity or carcinogenicity, and the role of host susceptibility and response (Cetta et al. 2007, 2009a).

In a genome-wide analysis of copy numbers in couples in which either husbands had been occupationally exposed to asbestos but did not have mesothelioma or spouses with mesothelioma who had not been occupationally exposed to asbestos, we reported a panel of differently expressed genes that could be responsible for a different inherited susceptibility. This panel of differently expressed genes sometimes included genes involved in the control of major histocompatibility systems, in the production of drug-metabolizing enzymes, or of X-ray repair or mismatch repair genes (Cetta F. Dhamo A, Zangari R, unpublished data).

Therefore, it is plausible that genetic polymorphisms in *GSTM1* and *GSTT1* may be part (if not the main determinant) of a panel of genes that define the individual susceptibility of some subjects to interact differently with a given environmental agent; this interaction would lead to cancer as a final outcome only in the susceptible individuals and not in others, even if the nonsusceptible individuals are more exposed to the same toxic or carcinogenic agent.

We suggest that the pathogenetic task (i.e., a better knowledge of the variable impact of the same toxic agent on different individuals) requires very specific and focused studies and not generic studies that combine skin and bone cancer grouped by the same code.

We suggest that studies rely less on the statistical power of numbers (cases and controls) and pay more attention to the homogeneity of populations, groups, or subgroups. These studies should focus not only on the biological but also on the pathophysiologic and pathogenetic plausibility of observed data; they should avoid mixing “apples and oranges.”

We suggest that researchers examine data carefully before they state that one event is influenced or not influenced by a causative or facilitating agent, namely when interactions between cause and effect are very complex and the causative relationship is not clear-cut (Cetta et al. 2007, 2009b). This is even more important when attempting to establish the relative impact of inherited or environmental factors in the occurrence of various types of cancers, each of which has its own peculiarity and wide variations, even within the range of tumors affecting the same organ or tissue (Cetta et al. 2007, 2009a).

In the future, there will be a major need for improved knowledge of causative and pathophysiologic mechanisms and for more strict adherence to this knowledge before designing epidemiologic or pathogenetic studies. These studies must rely more on the homogeneity of the enrolled population and on the direct cause and effect relationship between the causative agent and the expected outcome, and less on the number of enrolled subjects (if subjects are not appropriate for the scope of the study, their inclusion is potentially misleading. Panel studies in smaller but well-selected groups will give more useful information than large population studies that are missing the pathophysiologic and causative targets, in particular when large studies are based on too many inferences and/or extrapolations from old or inhomogeneous data.
